# Chemistry and Pharmacology of Cyperaceae Stilbenoids: A Review

**DOI:** 10.3390/molecules26092794

**Published:** 2021-05-10

**Authors:** Csilla Zsuzsanna Dávid, Judit Hohmann, Andrea Vasas

**Affiliations:** Department of Pharmacognosy, Interdisciplinary Excellence Centre, University of Szeged, Eötvös u. 6, 6720 Szeged, Hungary; davidzsuzsanna88@gmail.com (C.Z.D.); hohmann.judit@szte.hu (J.H.)

**Keywords:** *Carex*, *Cyperus*, *Scirpus*, resveratrol, stilbenoid dimers, oligostilbenoids, antiproliferative, antioxidant, anthelmintic activity

## Abstract

Cyperaceae is a cosmopolitan plant family with approx. 5000 species distributed worldwide. Several members of this family are used in traditional medicines for the treatment of different diseases. In the last few decades, constituents with great chemical diversity were isolated from sedges, and a wide range of biological activities were detected either for crude extracts or for pure compounds. Among the isolated compounds, phenolic derivatives are the most important, especially stilbenoids, and flavonoids. To date, more than 60 stilbenoids were isolated from 28 Cyperaceae species. Pharmacological investigation of Cyperaceae stilbenoids revealed that several compounds possess promising activities; mainly antiproliferative, antibacterial, antioxidant and anthelmintic effects. Isolation, synthesis and pharmacological investigation of stilbenes are increasing constantly. As Cyperaceae species are very good sources of a wide variety of stilbenes, and several of them occur in large amount worldwide, they are worthy for phytochemical and pharmacological investigations. Moreover, stilbenes are important from chemotaxonomical point of view, and they play a key role in plant defense mechanisms as well. This review summarizes the stilbenoids isolated from sedges, and their biological activities.

## 1. Introduction

Cyperaceae is a cosmopolitan family of monocot plants with approximately 100 genera and 5000 species. Previously, Cyperaceae and Poaceae have been regarded as related plant families [1], but recent cladistic analysis using molecular and morphological data indicates that the Cyperaceae family is more closely allied with the Juncaceae and Thurniaceae families [2]. Members of Cyperaceae, commonly called sedges, are grass-like flowering plants distributed throughout all the continents, except Antarctica. The diversity of genera is far greater in tropical regions [3]. The six largest genera with approximate numbers of species are *Carex* (*n* = 2000), *Cyperus* (*n* = 650), *Rhynchospora* (*n* = 250), and *Eleocharis*, *Fimbristylis* and *Scleria* each with about 200 species. Other notable genera are *Bulbostylis*, *Schoenus*, *Scirpus* and *Mapania* [3]. Many of the sedges are used in traditional medicines for the treatment of different diseases, e.g., stomach and bowel disorders, amenorrhoea, bronchitis, haematopoietic disorders, tumors, infectious diseases, pain and fever, diabetes, skin diseases, problems concerning the circulation, digestive, respiratory and reproductive organs.

Besides phenolic compounds, e.g., stilbenes, flavonoids, phenolic acids and phenylpropanoids, terpenoids, coumarins, quinones were also isolated from sedges [4]. However, the most significant constituents are stilbenoids. Most of the stilbenes of sedges are derivatives of resveratrol, which is probably the most extensively examined compound. Stilbenes and their derivatives have attracted increasing attention due to their diverse chemical structures and potential pharmacological applications because of their promising biological activities.

As Cyperaceae species are very good sources of a wide variety of stilbenes, and several of them occur in large amount worldwide, they are worthy for further phytochemical and pharmacological investigations. Furthermore, stilbenes are important from chemotaxonomical point of view, and they play a key role in plant defense mechanisms as well.

## 2. Cyperaceae Species

Cyperaceae species possess extremely diverse morphological characteristics; common features are the triangular stems (except for *Scirpus* genus) and the small, wind-pollinated flowers with the sepals or petals completely absent or reduced to scales, bristles, or hairs (“sedge spikelets”) maturing as lens-shaped or three-sided achenes or nutlets [5]. The largest genera in Cyperaceae family are *Carex* and *Cyperus*. Most species of the *Cyperus* genus are regarded as weeds, however, several *Carex* species are cultivated as ornamental plants. Holm et al. grouped weeds according to their weediness and listed four *Cyperus* species (*C. rotundus*, *C. esculentus*, *C. difformis*, *C. iria*) among the worst 33 weeds of the world, *C. rotundus* being the worst. In the rhizomes and tubers of *C. rotundus*, several compounds with allelopathic effects are produced that can inhibit the growth of other plants (e.g., cotton) [6]. Species of the *Carex* genus are widely distributed in the temperate zone, only a minority of the species being invasive and none of the species can be considered as an important agricultural weed. These features of *Carex* species are due to their restrictive habitat requirements, such as larger seeds, shorter propagation time, less propagation vectors, intolerance to harvesting or tillage, and the greater susceptibility to herbicides [6].

## 3. Traditional Uses of Cyperaceae Species

Although many Cyperaceae species are considered to be undesirable weeds, and their presence is of particular economic importance in some regions [7], sedges also have several traditional applications by humans; edible species are consumed as nutritional supplemental food, others are used for weaving household items and several species for their medicinal properties. *Cyperus papyrus* (papyrus) was first exploited by the ancient Egyptians for manufacturing paper. Besides, the ash of *C. papyrus* is applied for curing certain eye diseases, healing wounds and checking malignant ulcers from spreading [8]. Despite being the most troublesome weed in the world, *C. rotundus* (purple nutsedge) has been traditionally applied for several medicinal purposes, e.g., the root and rhizome for the treatment of stomach and bowel disorders (nausea, vomiting, spams, diarrhea, indigestion), since ancient times in India, China, Iran and Japan. Moreover, it is also used to cure diabetes, fever, inflammation, and malaria. The rhizome of *C. rotundus* is one of the major drugs incorporated into numerous Kampo preparations sold under the name of “Koubushi” (Rhizoma Cyperi) [9]. In the Ayurvedic medicine, *C. rotundus* is used for curing amenorrhoea, bronchitis, haematopoietic disorders, leprosy, spasms, diarrhoea and dysentery. The rhizome and the culm are recommended to treat malaria, cough, mental disorders (hysteria, insomnia, anxiety), loss of memory, dysuria and infertility in Europe and Asia [8,10]. In Asia, the leaves of *C. rotundus* were extensively used as a flavouring agent in foods. Its seeds are used in pickles, curries, and different bakery products [11]. The sap expressed from the widely consumed corms of *Eleocharis dulcis* (Chinese water chestnut) is considered to have antibiotic effect and also used in case of icterus [8]. In India, the aqueous extract of the root of *Carex baccans* (crimson seeded sedge) has been traditionally used as an anthelmintic by Jaintia tribes [12,13]. The infusion of *Remirea maritima* (beach star) rhizome is considered to have sudorific, diuretic, diaphoretic and anti-blenorrhagic effects and used mainly in Brazil [8]. The rhizome of *Scirpus fluviatilis* (river bulrush) is used as emmenagogue, galactogogue and antispasmodic in Japan and China, while the roots of *S. maritimus* (seaside bulrush) is applied as an adstringent and as a diuretic in China [14,15].

In order to emphasise the value of this interesting plant family, Simpson et al. compiled a comprehensive checklist, including 45 genera and 502 species/intraspecific taxa. Each entry encompasses the accepted and common names, distribution, habitat and economic or ethnobotanical significance [8].

## 4. Constituents of Cyperaceae Species

Although Cyperaceae is one of the largest monocot plant families possessing approx. 5000 species, only a small proportion of the species have been studied regarding their chemical composition and biological activities so far. According to the literature data, the majority of the isolated compounds are resveratrol oligomers or other stilbene derivatives, but flavonoids, phenolic acids, phenylpropanoids, coumarins, quinones and terpenoids (sesqui- and triterpenes, sterols) have also been identified from sedges [4,14].

The most extensively investigated species is probably *Cyperus rotundus*. According to the literature data, several metabolites (e.g., linolenic, myristic and stearic acids, alkaloids, flavonoids, furochromons, saponins, mono-, sesqui- and triterpenes, sitosterin, phenylpropanoids, phenolic acids and iridoids) have been identified from the tubers of *C. rotundus*. These metabolites are responsible for some of the therapeutic, and insecticidal, fungicidal effects [16,17,18]. The leaves and seeds of *C. rotundus* contain volatile oil rich in bactericidal and fungicidal compounds [19].

## 5. Occurrence of Stilbenes in Cyperaceae Species

### 5.1. Structural Characteristics of Stilbenes

**Figure 1:**
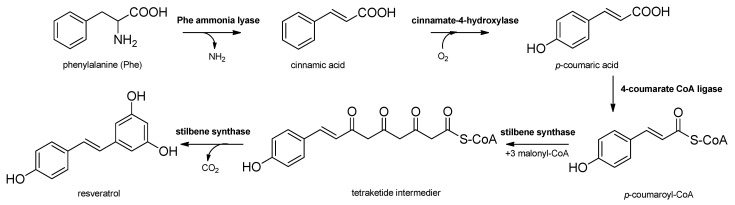
Biosynthesis of stilbenes.

**Figure 2:**
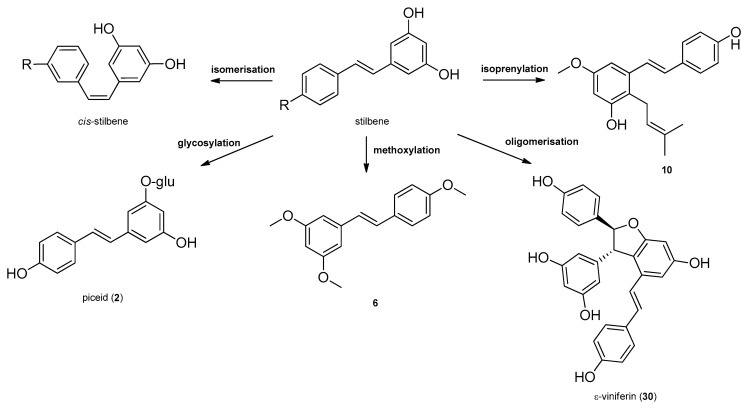
Most common modifications of stilbenes with examples from Cyperaceae species.

Stilbenes, a small class of plant phenolics are structurally characterized by a 1,2-diphenylethylene nucleus, and occur both as monomers, as well as dimers and complex oligomers. Since in monomeric stilbene aglycone’s skeleton the double bond between the two aromatic rings does not allow free rotation, there are only two possibilities of the configuration: the naturally more common and stable *trans*–(E), and the *cis*–(Z) configurations (Figure 1 and Figure 2). The two isomeric forms of stilbenes have different chemical characteristics and biological activities. The basic structure is frequently modified by several hydroxy groups, through which further substituents, among them methyl, isoprenyl groups, sugars and other residues can be attached to the stilbene backbone [20]. Oligomeric stilbenes are produced by oxidative coupling between homogeneous and heterogeneous monomers, that are linked by either C–C or C–O–C units. An 1,2-diaryl-dihydrobenzofuran skeleton with *trans*-oriented aryl rings is the most important framework in stilbene oligomers of this family, and it is considered to be biosynthesized by region and stereoselective pathways [9]. In 2008, Xiao et al. created a classification system, distributing stilbenes into six groups based on their structural characteristics, namely stilbenes, bibenzyls, bisbibenzyls, phenanthrenoids, stilbene oligomers and other stilbenoids [21]. In 2009, Shen et al. elaborated the classification of oligomeric stilbenes into four major groups based on the number of the connective bonds between the monomeric units [22].

### 5.2. Biosynthesis of Stilbenes

Stilbenes are synthetized constitutively in some plant tissues, like the bark, roots, fruits, and leaves. In other tissues, however, their synthesis can be induced by either biotic stresses, e.g., pathogen or herbivore attack, or abiotic stresses, e.g., wounding, UV irradiation and ozone [20]. Stilbenes are formed by the general phenylpropanoid pathway and occur in a number of heterogeneous and phylogenetically unrelated plant families, such as Cyperaceae, Dipterocarpaceae, Gnetaceae, Leguminosae, Polygonaceae, Vitaceae, etc. The initial reaction of the stilbene biosynthesis, catalyzed by the enzyme phenylalanine–ammonia lyase is the formation of cinnamic acid from phenylalanine. Subsequently, the hydroxylation of cinnamic acid by cinnamate 4-hydroxylase provides *p*-coumaric acid and 4-coumarate CoA ligase, followed by the formation of CoA esters of hydroxycinnamic acids. Stilbene synthase, the pivotal enzyme, catalyzes the biosynthesis of the stilbene backbone from three malonyl-CoA and one CoA-ester of a cinnamic acid derivative through a tetraketide intermediate [20,23]. Regarding the enzymatic features of stilbene biosynthesis, it has been reviewed in detail by Chong et al. in 2009 [23] (Figure 1). Stilbenes may then undergo different types of modifications, e.g., isomerization, methoxylation, glycosylation, isoprenylation and oligomerisation (Figure 2).

Stilbene oligomers may be classified biogenetically into two groups depending on the presence (group I) or lack (group II) of dihydrobenzofuran rings [24]. In group I, the dihydrobenzofuran ring has been attributed mainly to that of ε-viniferin (**30**) that has been isolated from the members of several plant families (e.g., Cyperaceae, Dipterocarpaceae, Fabaceae, Gnetaceae, and Vitaceae). Each family has a stereospecific biosynthetic pathway for the oxidative condensation of two stilbenoids, which definitely produces one enantiomer, as represented by (+)- and (‒)-ε-viniferin. ε-Viniferin (**30**) is a biogenetically important intermediate of stilbene oligomers in Cyperaceae species.

### 5.3. Isolation Procedures of Stilbenes from Cyperaceae Species

Stilbenes possess diverse structural characteristics, therefore, various separation methods have been applied to obtain these kind of metabolites from natural sources. According to the literature data, isolation of stilbenes is feasible from every part of Cyperaceae species. In some cases, stilbenes were isolated from the whole plant [25,26], however, other plant parts (rhizome or root [27,28], leaf [29], and seed [30]) were found to be a better source of stilbenes. The initial step of the isolation process is the extraction of the plant material. In most cases, pure methanol was used for the extraction, however, in some cases, ethyl acetate [29] or acetone [31] or the mixture of acetone‒methanol [32] were used. In other cases (e.g., *Carex distachya*), less polar solvents, like hexane [33] were applied. The crude extract is usually subjected to solvent-solvent partition with solvents of increasing polarity, among the most frequent ones were hexane, diethyl ether, ethyl acetate and dichloromethane. The following step is commonly a normal or reversed phase column chromatography (CC) performed on silica gel or Sephadex LH-20 gel using gradient elution. Typical solvent systems for normal phase CC are mixtures of hexane‒ethyl acetate, hexane‒acetone, chloroform‒methanol, dichloromethane‒methanol, dichloromethane‒ethyl acetate and dichloromethane‒acetone. Typical mobile phases applied for reversed phase CC were mixtures of acetonitrile‒water, methanol‒water and acetonitrile‒methanol‒water. After column chromatography, further chromatographic procedures are often required to obtain stilbenes in a pure form. These procedures include preparative thin layer chromatography (PTLC), and medium- and high-pressure liquid chromatography (MPLC/HPLC). In case of HPLC methods, reversed phase separations (mainly C18-columns [9,30,31,34,35,36]) are more frequently used than normal phase ones.

Stilbenes can be found in relatively high amounts in several Cyperaceae species, for instance the total content of this type of metabolites in the roots and rhizome of *Carex fedia* var. *miyabei* was estimated over 0.15% (*w/w* of fresh material) [28]. *Cyperus longus* is another good source of stilbenoids, its main constituents, scirpusins A (**31**) and B (**32**) could be detected in the rhizome at 0.028% and 0.008% (*w/w* of dried material), respectively [26]. In case of *Carex pumila*, the main constituent was miyabenol A (**64**) presented at 0.23% (*w/w* of dried material) in the plant [27].

### 5.4. Stilbenes Isolated from Cyperaceae Species

**Figure 3:**
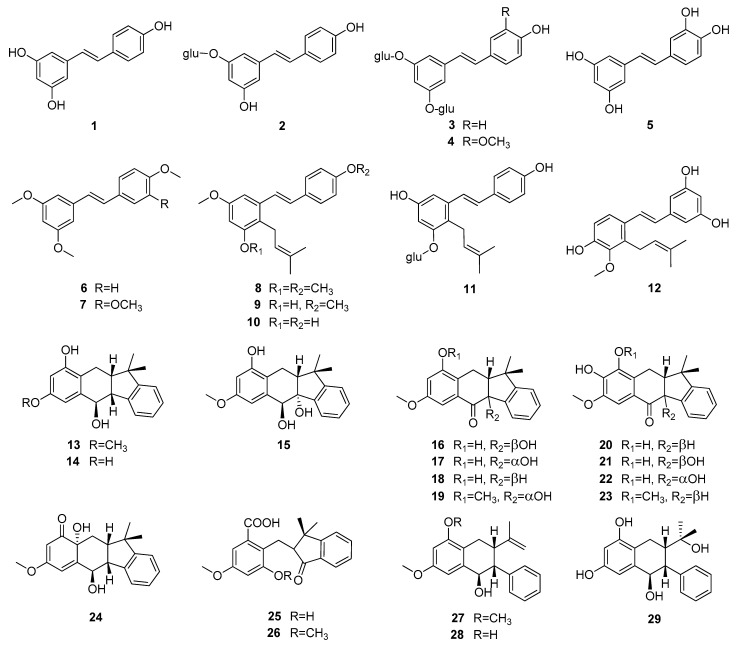
Stilbene monomers isolated from Cyperaceae species (glu = glucose).

**Figure 4:**
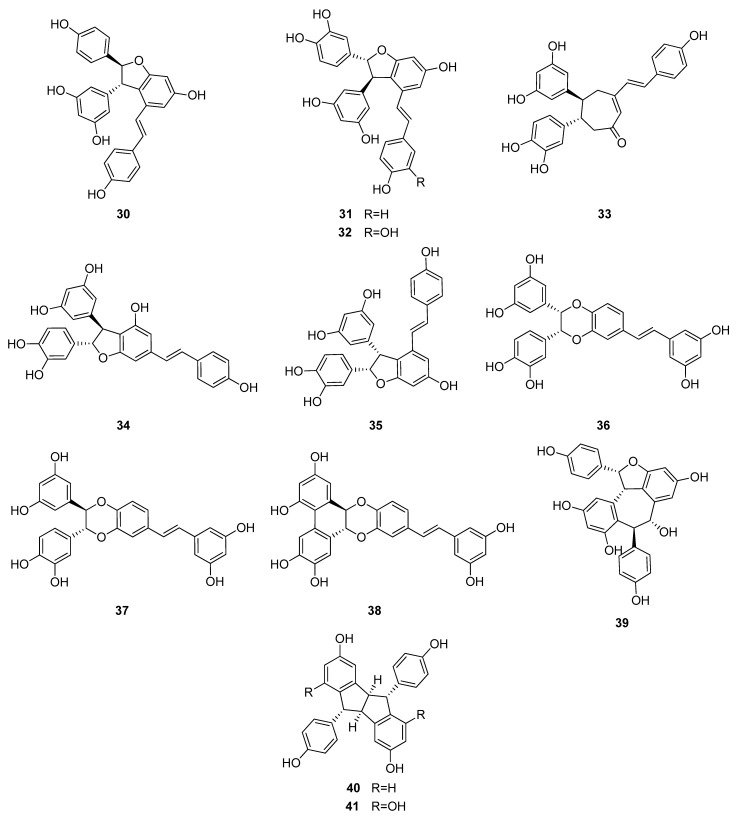
Dimeric stilbenes isolated from Cyperaceae species.

**Figure 5:**
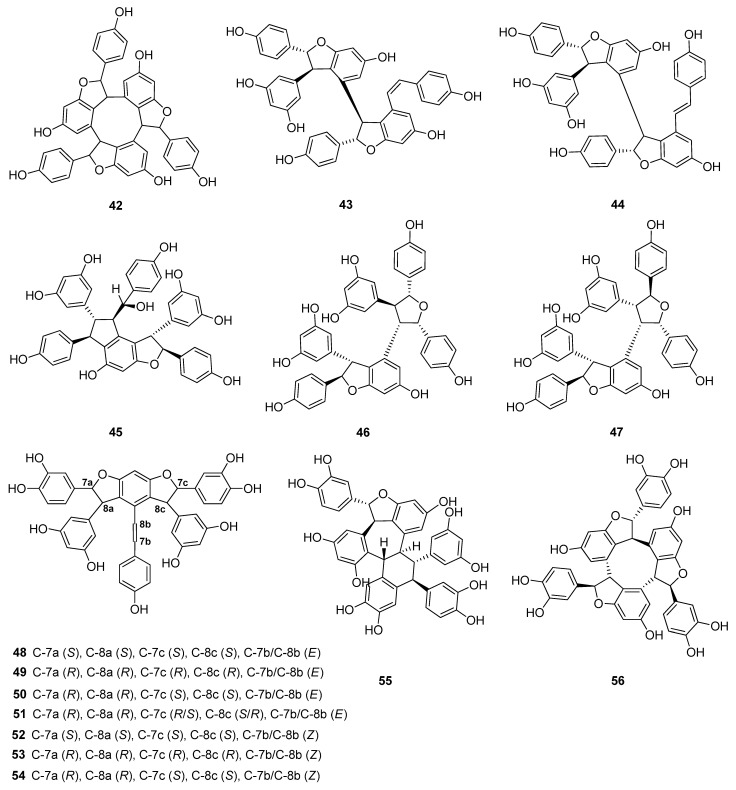
Stilbenoid trimers isolated from Cyperaceae species.

**Figure 6:**
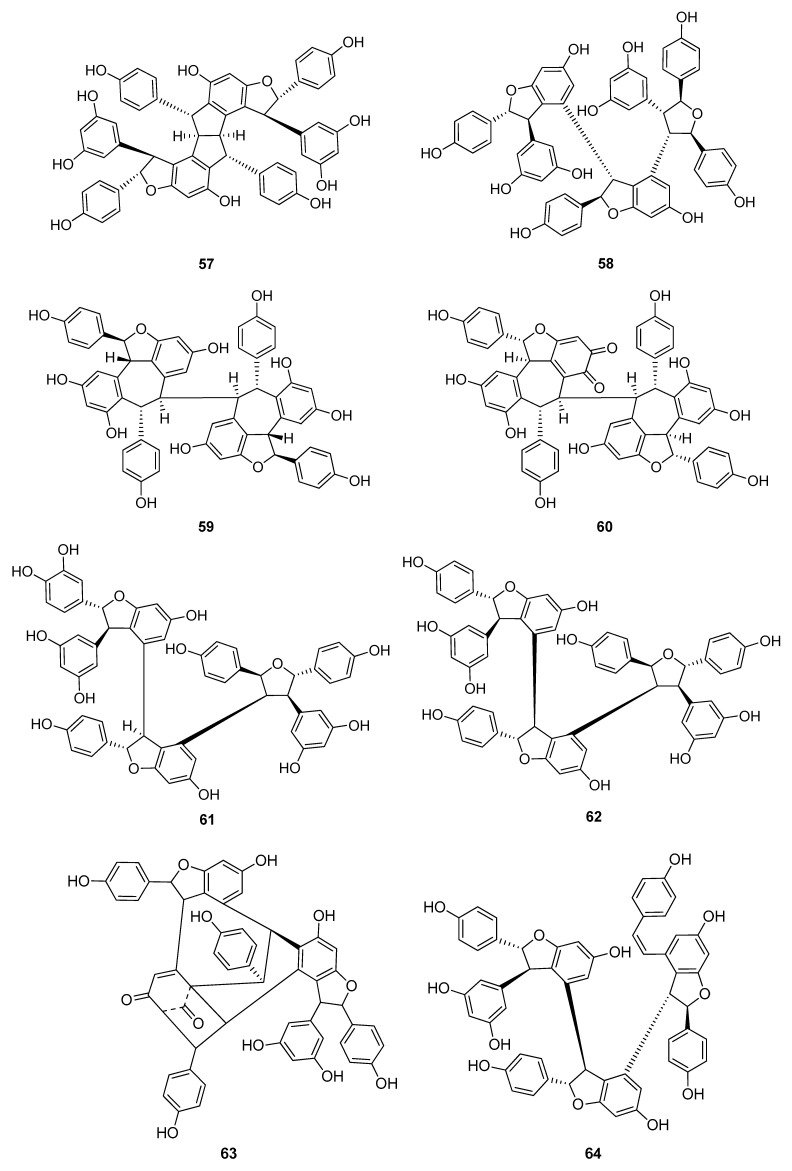

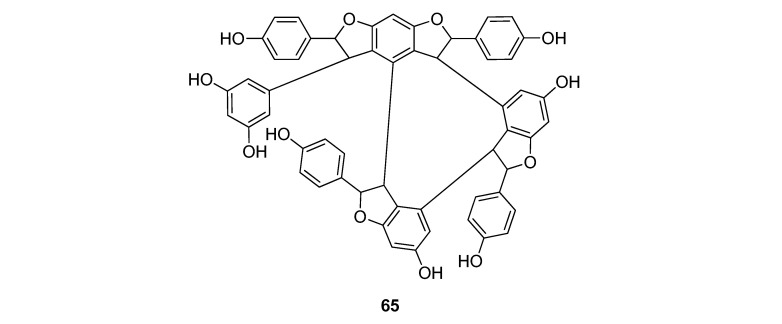
Stilbenoid tetramers isolated from Cyperaceae species.

Up to now 65 stilbenes have been isolated from different Cyperaceae species; *Carex* (*n* = 17), *Cyperus* (*n* = 4), *Carpha* (*n* = 1), *Kobresia* (*n* = 1), *Scirpus* (*n* = 4) and *Schoenus* (*n* = 1) (Figure 3, Figure 4, Figure 5 and Figure 6, Table 1). Among the identified compounds, there are 29 monomers (compounds **1**–**29**), 12 dimers (compounds **30**–**41**), 15 trimers (compounds **42**–**56**), and nine tetramers (compounds **57**–**65**). Diversity of stilbenes is due to the number and type of the connecting monomers and substituents resulting in dimers, trimers and tetramers. Monomers are joining to each other via forming one or more tetrahydrofuran rings.

Around 45% of stilbenes reported from sedges are monomers (Figure 1). All of them are substituted, most frequently at C-3, C-5 and C-3′, but substitution can be found also at other places. The most common substituent is hydroxy group, through which one (**11**, **41**) or two (**3**, **4**) glucose molecules can connect to the basic structure. Methoxy group is the second most common substituent, linking mainly at C-2 and C-4. Prenyl-substituted stilbenes were isolated only in monomeric form, from *Scirpus holoschoenus* (**8**–**10**), *Schoenus nigricans* (**8**–**10**), *Carex vulpinoidea* (**11**) and *Carpha glomerata* (**12**) [37,38,39,40]. Prenyl group joined to the skeleton at C-2 in all cases. Carexanes **13**–**29**, isolated from *Carex distachya*, originate by the prenylation and successive cyclization of a stilbene precursor [29,33,41,42,43,44]. Compounds **25** and **26** are carbonyl substituted. The most common monomers are *trans*-resveratrol (**1**) and piceatannol (**5**). The only difference between the compounds is the presence of an extra hydroxy group in **5**. Compound **1** was isolated from *Carex baccans*, *C. dimorpholepis*, *C. pumila*, *Cyperus longus*, *C. stoloniferus* and *Scirpus maritimus*, while compound **5** from *Carex apressa* var. *virgata*, *Cyperus longus*, *Scirpus californicus*, and *S. maritimus*.

*trans*-Resveratrol (**1**) is the common constituent of most of the oligomers isolated from sedges. Scirpusins A (**31**) and B (**32**) are abundant stilbene dimers in *Scirpus* and *Cyperus* species [14,15,26,45] (Figure 4). Scirpusin A (**31**) is the heterodimer of *trans*-resveratrol (**1**) and piceatannol (**5**), while scirpusin B (**32**) is evolved through the connection of two resveratrol units. ε-Viniferin (**30**), a resveratrol dimer, occurs mainly in *Carex* species (*C. appressa* var. *virgata*, *C. fedia* var. *miyabei*, *C. kobomugi*, and *C. pumila*) but it is a biogenetically important intermediate of stilbene oligomers in Cyperaceae species. Longusone A (**33**), isolated from *Cyperus longus*, is the only norstilbene dimer identified from Cyperaceae family; it contains a tropilene skeleton [26].

(+)-α-Viniferin (**42**) is a common stilbene trimer in Cyperaceae species (*Carex glauca*, *C. gynandra* [31], *C. humilis* [46], and *C. baccans* [47]), originating from the condensation of 3 resveratrol monomers (Figure 5).

The stilbenoids, isolated from *C. fedia* var. *miyabei* are derivatives of resveratrol (**1**); **30** is the dimer of two units of **1**, miyabenol C (**43**) is a trimer and miyabenols A (**64**) and B (**65**) are tetramers (Figure 6). **65** is formed from **64** via intramolecular oxidative cyclization involving a hydroxy group and a double bond in the *trans*-stilbene residue, while **43** is a biogenetic intermediate between **30** and **64** and **65**. The total content of these metabolites in the underground part of the plant was estimated over 0.15% (*w/w* of fresh plant), miyabenol A (**64**) being the predominant stilbenoid with more than 0.1% [28].

Besides miyabenols A and B (**64**, **65**, tetramers), in cases of kobophenols A and B (**62**, **63**, tetramers), it is also ε-viniferin (**30**) serving as a building block compound. Compound **62** was isolated from *Carex kobomugi* and it has a unique 2,3,4,5-tetraaryl-tetrahydrofuran skeleton [48,49]. Kobophenol B (**63**), isolated from *Carex gynandra* [31], *Carex pendula* [30], and *Carex pumila* [50], has an unprecedented polycyclic structure [32]. In plants, that synthesize kobophenol B (**63**) it is the main stilbenoid constituent; its amount was between 0.1–1.27% (*w/w* of dried plant material). This compound is originated by the condensation of two (–)-ε-viniferin (**30**) units. The resveratrol oligomers (3 trimer and 4 tetramers) nepalensinols A‒G (**45**–**47**, **57**–**60**) were isolated from the stems of *Kobresia nepalensis* [36,51].

To date, most compounds (*n* = 18) were isolated from Carex distachya; carexanes (**13**–**29**) are specific from chemical point of view, as they have a rare tetracyclic structure. Such compounds were determined only from this species. Cyperus longus and Cyperus rotundus are also remarkable sources of stilbenes, from which 10 (**1**, **5**, **31**–**37**, **40**) and 13 (**2**, **31**, **32**, **37**, **48**–**56**) compounds were isolated, respectively. The isolated stilbenes seem to be stable, only in cases of cis-miyabenol A (**64**) and cis-miyabenol C (**43**) was mentioned that if they are exposed to light, they isomerize to the trans-isomers [30].

**Table 1:** Stilbenoids of Cyperaceae species.

Species	Compound	Ref.
*Carex appressa* var. *virgata*	**3**, piceatannol (**5**), ε-viniferin (**30**), virgatanol (**39**)	[25]
*Carex baccans*	*trans*-resveratrol (**1**), α-viniferin (**42**)	[13,47,52]
*Carex buchananii*	kobophenol A (**62**)	[34]
*Carex capillacea*	longusol B (**35**), (*E*)-miyabenol A (**64**)	[34]
*Carex cuprina*	carexinol A (**61**), kobophenol A (**62**)	[25,34]
*Carex dimorpholepis*	*trans*-resveratrol (**1**)	[53]
*Carex distachya*	**3**, **4**, carexane A (**13**), carexane M (**14**), carexane N (**15**), carexane B (**16**), carexane C (**17**), carexane D (**18**), carexane J (**19**), carexane E (**20**), carexane F (**21**), carexane G (**22**), carexane K (**23**), distachyasin (**24**), carexane H (**25**), carexane L (**26**), carexane I (**27**), carexane P (*28*), carexane O (**29**), pallidol (**40**), 41	[29,33,41,42,43,54]
*Carex fedia* var. *miyabei*	ε-viniferin (**30**), *trans*-miyabenol C (**44**), (*E*)-miyabenol A (**64**), miyabenol B (**65**)	[28]
*Carex folliculata*	pallidol (**40**), kobophenol A (**62**)	[31,55]
*Carex glauca*	pallidol (**40**), α-viniferin (**42**), *cis*-miyabenol C (**43**)	[34,54]
*Carex gynandra*	pallidol (**40**), α-viniferin (**42**), *trans*-miyabenol C (**44**), kobophenol B (**63**)	[31]
*Carex hirta*	**3**, (*E*)-miyabenol A (**64**)	[34]
*Carex humilis*	α-viniferin (**42**)	[46,56]
*Carex kobomugi*	ε-viniferin (**30**), *trans*-miyabenol C (**44**), kobophenol A (**62**)	[48,49]
*Carex pendula*	*cis*-miyabenol C (**43**), kobophenol B (**63**), (*E*)-miyabenol A (**64**)	[30]
*Carex pumila*	*trans*-resveratrol (**1**), ε-viniferin (**30**), *trans*-miyabenol C (**44**), kobophenol B (**63**), (*E*)-miyabenol A (**64**)	[27,32,50]
*Carex vulpinoidea*	vulpinoideol A (**11**)	[39]
*Carpha glomerata*	carphaben (**12**)	[40]
*Cyperus eragrostis*	scirpusin B (**32**), cyperusphenol B (**55**)	[25]
*Cyperus longus*	*trans*-resveratrol (**1**), piceatannol (**5**), scirpusin A (**31**), scirpusin B (**32**), longusone A (**33**), longusol A (**34**), longusol B (**35**), longusol C (**36**), cassigarol E (**37**), cassigarol G (**38**), pallidol (**40**)	[26]
*Cyperus rotundus*	piceid (**2**), scirpusin A (**31**), scirpusin B (**32**), cassigarol E (**37**), (+)-(*E*)-cyperusphenol A (**48**), (–)-(*E*)-cyperusphenol A (**49**), (*E*)-mesocyperusphenol A (**50**), (*E*)-cyperusphenol C (**51**), (+)-(*Z*)-cyperusphenol A (**52**), (–)-(*Z*)-cyperusphenol A (**53**), (*Z*)-mesocyperusphenol A (**54**), cyperusphenol B (**55**), cyperusphenol D (**56**)	[9,35]
*Cyperus stoloniferus*	*trans*-resveratrol (**1**), piceatannol (**5**)	[57]
*Kobresia nepalensis*	nepalensinol A (**45**), nepalensinol C (**46**), nepalensinol D (**47**), nepalensinol B (**57**), nepalensinol E (**58**), nepalensinol F (**59**), nepalensinol G (**60**)	[36,51]
*Scirpus californicus*	piceatannol (**5**), scirpusin A (**31**), scirpusin B (**32**)	[45]
*Scirpus fluviatilis*	*trans*-resveratrol (**1**), piceatannol (**5**), scirpusin A (**31**), scirpusin B (**32**)	[14]
*Scirpus holoschoenus*	**6**, **8**	[37]
*Scirpus maritimus*	*trans*-resveratrol (**1**), ε-viniferin (**30**), scirpusin A (**31**), scirpusin B (**32**)	[15]
*Schoenus nigricans*	**6**, **7**, **9**, **10**	[38]

## 6. Pharmacological Activities of Cyperaceae Species and the Isolated Compounds

Stilbenes and their derivatives have attracted increasing attention due to their diverse biological activities and potential pharmacological applications. Some of these secondary metabolites have been recognized as phyotoalexins and associated with the defense mechanisms of plants as they are produced after infection by pathogens or exposure to UV radiation and present antifungal activities. Probably, the most extensively investigated compound is resveratrol (*trans*-3,5,4′-trihydroxystilbene, **1**), of which over 2000 papers have been published. Resveratrol (**1**) has gained attention when being associated with “the French paradox”, the well-documented phenomenon of the relatively low incidence of coronary heart disease despite high dietary intake of saturated fats in southern France, that can be explained by the protective effect of moderate wine consumption [58,59]. It has been proven by several studies that the favorable cardiovascular effect of red wine is mainly due to its content of phenolic compounds, especially resveratrol. Since then, numerous biological activities of resveratrol (**1**) have been reported, among them antioxidant, anticancer, anti-inflammatory, antidiabetic, cardioprotective, antiaging effects and it was proven to be a phytoestrogen as well [60].

Pharmacokinetic studies of resveratrol (**1**) indicated that during circulation in the plasma it is extensively metabolized and its oral bioavailability is close to zero, due to factors such as limited absorption, limited chemical stability, and degradation by intestinal microflora and intestinal enzymes [61]. The major metabolites identified in the plasma and urine by metabolic studies are resveratrol glucuronides and sulphates [62]. In case of the dimer ε-viniferin (**30**), it was observed that its intestinal absorption rate is low and negligible compared to that of resveratrol [61]. However, these findings are controversial with the multitude biological effects of resveratrol (**1**) confirmed in vivo. This can be explained by the capability of **1** to bind to transport proteins, like human serum albumin and lipoproteins forming complexes, in which resveratrol is more stable and can enter into different tissues as well [63,64,65]. Another possible explanation is that the concentration of the glucuronide and sulphate type metabolites in the blood is higher than the initial concentration of **1**, proposing that resveratrol might be released locally in the target organ/tissue from these metabolites [60,63]. The “broad spectrum” of biological activities is likely a reflection of the intrinsic reactivity of the trihydroxylated stilbene **1** as a redox-active molecule. Mounting evidence suggests that resveratrol and its oligomers exert their effects via interference with signal transduction cascades and epigenetic pathways rather than direct inhibition of enzymes designated for specific purposes [66,67].

Besides resveratrol, other monomeric (e.g., piceatannol, combretastatin A-4, etc.) and oligomeric (e.g., α-viniferin, hopeaphenol A, miyabenol C and kobophenol B) stilbenes with promising biological activities have also been isolated from natural sources in recent years. An enormous number of studies have been undertaken to define their diverse structures and biological activities. As a result, there are several review articles summarizing the phytochemistry and pharmacology of naturally occurring stilbenes [21,22,68,69,70,71,72]. Stilbenes possess a wide range of multi-faceted biological activities, among them antitumor, antioxidant, antiplatelet, antimicrobial, antidiabetic, anti-inflammatory, neuro-, cardio- and hepatoprotective, spasmolytic, ecdysteroid antagonist and tyrosinase inhibitory activities. Therefore, stilbenes are of significant interest for researchers in the process of developing new drugs and medicines [73].

The most investigated Cyperaceae species is *C. rotundus*. Based on the pharmacological studies performed with this plant, its tuber and rhizome possess anti-diarrheal, antioxidant, anti-inflammatory, anticonvulsive, antipyretic, antifungal, antidiabetic, antimalarial, antihyperlipidemic, antibacterial, antiviral, antiproliferative, cardio protective and wound healing effects [74,75,76,77,78].

In this part of the review, those Cyperaceae species are discussed from which stilbenoids were isolated and their pharmacological activities were also tested (Table 2).

### 6.1. Promising Effects Regarding Human Health

#### 6.1.1. Antitumor Activity

Resveratrol (**1**) possesses a wide range of biological effects, including suppressing the growth of a wide variety of tumor cells (e.g., breast, prostate, hepatic, skin, lung, colon, and pancreas cells) through inhibition of DNA polymerase and ribonucleotide reductase, and by inducing cell cycle arrest or apoptosis initiating caspase-8-dependent or caspase-9-dependent pathways [79]. Resveratrol (**1**) was found to be a natural killer (NK) cell activator; it had a synergistic effect with IL-2 on enhancing the cytolytic activity of NK cells and activated Akt by regulating Mammalian Target of Rapamycin Complex 2 (mTORC2) via phosphatase and tensin homolog (PTEN) and ribosomal protein S6 kinase beta-1 (S6K1) [80]. Moreover, it was observed that resveratrol (**1**) increases the susceptibility of aggressive cancer cells to T-cell-mediated cell death via disrupting the glycosylation and dimerization of programmed death ligand-1 (PD-L1) and impeding the PD-1 interaction surface of PD-L1 [81].

The antiproliferative effect of stilbenoids (**40**, **42**, **44**, **62**, and **63**), isolated from *C. folliculata* and *C. gynandra*, together with resveratrol (**1**) were tested against human colon tumor cell lines (HCT-116, HT-29, Caco-2) and on normal human colon (CCD-18Co) cells. Among them, α-viniferin (**42**) was the most active against the colon cancer cells with IC_50_ values of 6.6 µM (HCT-116), 32.6 µM (HT-29), and 16.1 µM (Caco-2). It was >2-fold more effective against cancer cells, compared to normal colon cells (IC_50_ 40.0 µM). Moreover, compound **42** did not induce apoptosis at 20 µM but arrested cell cycle for the colon cancer but not the normal colon cells [31].

Nepalensinols A‒G (**45**–**47**, **57**–**60**) were tested for their inhibitory activity [IC_50_ values 0.30 µM (**45**), 0.02 µM (**57**), 7.0 µM (**46**), 14.8 µM (**47**), 11.7 µM (**58**), 5.5 µM (**59**)] against human DNA topoisomerase II. Compound **57** exhibited the most potent activity, which was 3 × 10^3^ times stronger than that of etoposide. Nepalensinol G (**60**) was proven to be inactive in this test system. Etoposide (IC_50_ = 70 µM) and daunorubicin (IC_50_ = 9.1 µM) were used as positive controls [36,51].

The antiproliferative effect of oligostilbenoids, isolated from the rhizome of *C. rotundus*, were demonstrated against human T-cell leukemia Jurkat cells. Among the tested compounds, the racemates (**48** and **49**), **50**, **55**, and **56** had marked inhibitory activity, with IC_50_ values 27.4, 40.5, 26.4 and 26.3 µM, respectively. The apoptotic effect of cyperousphenol D (**56**) was mediated mainly by affecting the activation of caspase-3 [9].

#### 6.1.2. Antioxidant Activity

The incidence of tumor increases after exposure to free radicals. The antioxidant capacity of free radical scavengers is responsible for their antimutagenic effects [82]. Therefore, the antiradical activities of compounds could also be worthy for investigation [83].

The methanol extract of *C. longus* showed DPPH radical scavenging activity at SC_50_ = 22 µg/mL (the concentration required for a 50% reduction of 40 µM DPPH radical). Among the isolated compounds, *trans*-scirpusin B (**32**) was found to possess the most potent DPPH radical scavenging activity (SC_50_ = 2.8 µM), and the scavenging activity of stilbene dimers [8.2 µM (**31**), 2.8 µM (**32**) 4.6 µM (**33**), 9.3 µM (**34**), 4.3 µM (**35**), 5.0 µM (**36**), 3.2 µM (**37**), and 4.5 µM (**38**)] were stronger than those of monomers (24 µM for **1**, and 11 µM for **5**), except for pallidol (**40**, IC_50_ = 29 µM) [26]. Among the compounds isolated from the rhizome of *C. rotundus*, cyperusphenol B (**55**) was the most active (65% at 5 µg/mL) in scavenging free radicals in DPPH assay. The other components showed 58% (**50**), 49% (**48** and **49**), 47% (**56**) and 37% (**31**) free radical scavenging activity. All compounds, with the exception of **31** possessed higher activity than the positive control ascorbic acid (46%) [9].

The antioxidant activity of methanolic root extract of *Carex distachya* was measured by its capability to scavenge the DPPH radical (IC_50_ value 4.2 µg/mL) and it was comparable to those of ascorbic acid, α-tocopherol and butylated hydroxytoluene (BHT) (IC_50_ values 4.3, 5.1, and 3.9 µg/mL, respectively). This high antioxidant power of the extract is due, at least partly to its stilbenoids, **3**, **4**, and **41**. Resveratrol derivatives **3** and **4**, showed a strong nitric oxide radical scavenging capacity (>80% for both, 58.5% for ascorbic acid and α-tocopherol, and 62.2% for BHT, respectively) [54]. Distachyasin (**24**) showed radical scavenging activity against superoxide radical for 60% at 0.5 mg/mL, hydrogen peroxide at 0.1 and 0.2 mg/mL by 32% and 39%, and NO radical at 0.5 mg/mL by 10.7%. Moreover, **24** inhibited the formation of reactive oxygen species to thiobarbituric acid over 59.0% at 0.5 mg/mL [43].

Piceatannol (**5**), scirpusins A (**31**) and B (**32**), isolated from *S. californicus*, showed xanthine oxidase inhibitory activity (IC_50_ values 3.9, 3.6 and 6.0 µM, respectively) [45]. Piceatannol (**5**) was found to interact with several molecular targets when its antioxidant activity was investigated. It inhibited *Propionibacterium acnes*-induced HaCaT cell proliferation, promoted the nuclear translocation and target gene transcription of the antioxidant transcription factor nuclear factor erythroid 2-related factor 2 (Nrf2), and reduced the level of intracellular reactive oxygen species (ROS) [84].

#### 6.1.3. Anti-Inflammatory Activity

The acidic and amphiphilic character of stilbenoids causes their enrichment in biomembranes, where many of their targets occur (COX, 5-LOX, protein kinase B) [85]. Anti-inflammatory and antioxidant activities stand behind nearly all of the other positive pharmacological effects of stilbenoids. When compared to oligomeric stilbenoids the monomers have been studied much more intensely. This is probably related to their higher abundance in nature and simple structure enabling their easier identification and further structural modification towards novel derivatives [86].

The anti-inflammatory activity of resveratrol (**1**) is associated with its ability to inhibit COX-1 and COX-2 activity [87]. It has also been demonstrated that resveratrol inhibits the activity of transcription factors NF-κB (nuclear factor kappa B) and AP-1 (activator protein-1), both of which directly regulates the activity of cyclooxygenases as well as inducible nitric oxide synthase [88]. Moreover, it inhibits the induced production of pro-inflammatory cytokines, such as TNFα (tumor necrosis factor α), IL-1β, IL-6 or IL-8 [89,90] and matrix metallopeptidases MMP-2, MMP-3, MMP-9 and MMP-13 [89,91].

It has been shown that resveratrol derivatives with additional *ortho*-hydroxy group exhibit more potent antioxidant and anti-inflammatory effects in vitro due to the ability to form semiquinone radical. For example, piceatannol (**5**) was reported to be about 400 times more selective towards the inhibition of COX-2 enzyme than resveratrol [92], and it activated more potently heme oxygenase-1 (HO-1) enzyme as well [93]. Piceatannol (**5**) was found to impede the nuclear translocation of p65 [a subunit of nuclear factor kappa B (NF-κB)] and the secretion of proinflammatory cytokines, including interleukin-6 (IL-6), TNF-α and interleukin-8 (IL-8) as well as it inhibited the inflammatory NF-κB pathway [84].

α-Viniferin (**42**), isolated from *C. humilis* was tested for its inhibition on cyclooxygenase activity of prostaglandin H_2_ synthase. The compound exhibited a dose-dependent inhibitory activity (IC_50_ 7 µM) which was comparable to the positive control indomethacin (IC_50_ 5 µM). This effect was about 3- to 4-fold stronger than that of resveratrol (IC_50_ 25 µM) [46]. Other anti-inflammatory mechanisms of actions of α-viniferin (**42**) were also determined. Chung et al. reported that **42** down-regulates signal transducer and activation of transcription-1 (STAT-1)-inducible inflammatory genes through impeding extracellular signal-regulated kinase (ERK)-mediated STAT-1 activation in interferon-γ (IFN-γ)–stimulated macrophages [94], while Dilshara et al. found **42** to be effective in lipopolysaccharide (LPS)-induced inflammation via suppressing the production of nitric oxide (NO) and prostaglandin E2 (PGE2) and inhibiting the expression of iNOS and COX-2 in LPS-treated BV2 microglial cells through suppression of PI3K/Akt-dependent NF-κB activation and increasing Nrf2-mediated HO-1 expression [95].

Carexanes **16**, **22**, and **27** were able to enhance the antioxidant response of HspB-transfected human gastric epithelial (AGS) cells. Among them, carexane I (**27**) proved to be the most active; it was able to reduce Keap-1 gene expression and induce NQO1 gene expression in AGS cells. Moreover, it reduced COX-2 gene expression in HspB-transfected AGS cells [42].

#### 6.1.4. Antiallergic Activity

The methanol extract of *C. longus* showed antiallergic effect on ear passive cutaneous anaphylaxis (PCA) reactions in mice at a dose of 500 mg/kg (75% inhibition), per os. Some of the stilbenoids (**1**, **5**, **36**, **37** and **38**), isolated from the plant inhibited the release of β-hexosaminidase (IC_50_ values 17 µM, 24 µM, 96 µM, 84 µM, and 84 µM, respectively), a marker of antigen-induced degranulation, in rat basophilic leukaemia (RBL-2H3) cells. These compounds proved to be more potent than the positive controls tranilast (IC_50_ = 112 µM) and ketotifen fumarate (IC_50_ = 176 µM). Moreover, monomers (**1** and **5**) showed higher activities than the dimers **36**, **37** and **38** [26].

#### 6.1.5. Antimicrobial Activity

Miyabenol A (64), a metabolite of *C. fedia* var. *miyabei*, showed antimicrobial activities against *Staphylococcus aureus* and *Bacillus subtilis* at a level of less than 10 µg/8 mm diameter paper disc [28].

The antitubercular activity of α-viniferin (**42**), isolated from *C. humilis*, were tested against drug-susceptible and –resistant strains of *Mycobacterium tuberculosis*. The compound showed antibacterial effect against both strains at MIC_50_s of 4.6 µM in culture broth medium and MIC_50_s of 2.3–4.6 µM inside macrophages and pneumocytes. An additive effect and partial synergy were observed against *M. tuberculosis* H37Rv, when it was applied in combination with streptomycin and ethambutol. α-Viniferin (**42**) did not show cytotoxicity in any of the cell lines tested up to a concentration of 147 µM, proving its selectivity index of > 32. Moreover, α-viniferin (**42**) inhibited the proliferation of methicillin-susceptible *Staphylococcus aureus* (MSSA), methicillin-resistant *S. aureus* (MRSA) and methicillin-resistant *S. epidermidis* (MRSE) with a MIC values of 9.2–18.4 µM [56].

The resveratrol dimer ε-viniferin (**30**) inhibited the biofilm formation of enterohemorrhagic *Escherichia coli* O157:H7 (EHEC) at 10 µg/mL by 98% without affecting planktonic growth [50]. The extract of *Carex dimorpholepis*, and its constituent resveratrol (**1**) also possessed anti-biofilm activity against EHEC at 10 µg/mL. Moreover, the extract decreased the adhesion of EHEC cells to human colonic epithelial (HT-29) cells without affecting the viability of these cells [53].

#### 6.1.6. Anthelmintic Activity

*C. baccans* has been traditionally used in Northeast India to get rid of intestinal worm infections. In an experiment, in vivo cestocidal activity of root tuber extract of the plant and its stilbene constituent resveratrol (**1**) was tested against the zoonotic cestode *Hymenolepis diminuta*. The activity was determined by monitoring the egg per gram (EPG) counts in feces of different treat groups of rats. At 50 mg/kg of plant extract, and 4.56 mg/kg body weight of resveratrol (**1**), both possessed significant anthelmintic effect against the worm. Both reduced EPG count (56.0% and 46.1%) and decreased worm burden by 44.3% and 31.0%, respectively. Praziquantel was used as a positive control [13]. The anthelmintic effect of resveratrol (**1**) and α-viniferin (**42**) was evaluated against *Raillietina echinobothrida* in comparison to the reference drug praziquantel. It was observed that the parasites ceased movement at 9.4, 11.4, and 0.2 h followed by death at 23.7, 34.2, and 1.9 h, respectively. Moreover, a significant decrease in the activity of acetylcholinesterase (46.1% and 65.9%) and nitric oxide synthase (61.2% and 55.0%) were detected in comparison with the controls Nω-nitro-l-arginine (29.6%) and pyridostigmine (63.6%). Therefore, it can be concluded that the anthelmintic effect of these compounds is mediated through inhibition of two vital enzymes [47].

#### 6.1.7. Antidiabetic Activity

Numerous studies on diabetic rats revealed the anti-hyperglycemic action of resveratrol (**1**). Among different beneficial effects of resveratrol found in diabetes, the ability of this compound to reduce hyperglycemia seems to be the best documented. The anti-hyperglycemic action of resveratrol was demonstrated in obese rodents and in two animal models of diabetes: in rats with streptozotocin induced diabetes or with streptozotocin-nicotinamide-induced diabetes. Some studies also revealed that administration of resveratrol (**1**) to diabetic rats resulted in diminished levels of glycosylated hemoglobin (HbA1C), which reflects the prolonged reduction of glycaemia [96,97]. The anti-hyperglycemic effect of resveratrol observed in diabetic animals is thought to result from its stimulatory action on intracellular glucose transport through increased expression of the insulin-dependent glucose transporter GLUT4 [98].

The ethyl acetate extract of *C. baccans* (EAC) and its main stilbenoid constituent (+)-α-viniferin (**42**) inhibited α-glucosidase [IC_50_ values 31.8 µg/mL (EAC), and 6.8 µg/mL (**42**)] and α-amylase [IC_50_ values 421.1 µg/mL (EAC), and 282.9 µg/mL (**42**)] enzymes [52].

Piceatannol (**5**) promoted glucose uptake, AMPK phosphorylation and GLUT4 translocation to plasma membrane in L6 myocytes in vitro, and it decreased the rises in blood glucose levels at early stages and improved the impaired glucose tolerance at late stages in vivo in type 2 diabetic model in mice [99].

#### 6.1.8. Vasorelaxant Activity

Arginase catalyzes hydrolysis of L-arginine to L-ornithine and urea and plays an important role in the ammonia detoxification in mammals. By substrate competition, it also plays a crucial role in the bioavailability of L-arginine for nitric oxide synthase (NOS). The result of this competition is the decrease of nitric oxide (NO) production and the increase of L-ornithine production. This latter is converted into polyamines or proline that can promote cell proliferation and collagen production, resulting in various health problems, in particular at the cardiovascular level. Compounds with arginase inhibitory activity may have use to treat e.g., microbial or parasitic infections, cancers and inflammatory or cardiovascular diseases [100].

Resveratrol (**1**) could prevent the hypoxia-induced increased arginase activity, arginase II mRNA and protein expression, and proliferation in hPASMC (human pulmonary artery smooth muscle cell). It also prevented the hypoxia-induced Akt activation, and attenuated chronic hypoxia-induced RVH (right ventricular hypertrophy) in neonatal rats by normalization of RV/(LV S) ratios [101].

Piceatannol (**5**), ε-viniferin (**30**), scirpusin B (**32**), cyperusphenol B (**55**) and carexinol A (**61**) significantly inhibited the activity of arginase enzyme with IC_50_ values of 12.6, 27.8, 22.6, 12.2 and 25.3 µM, respectively; therefore, increasing the bioavailability of NO and resulting in vasorelaxant activity [25].

### 6.2. Effects Useful for Agriculture

#### 6.2.1. Effect on Plant Growth

Plants can response to different infections in several ways. One of the best-known and longest-studied one is the induced accumulation of antimicrobial, low-molecule-weight secondary metabolites known as phytoalexins. Phytoalexins are chemically diverse molecules, including simple phenylpropanoid derivatives, flavonoids and isoflavonoids, sesquiterpenes and polyketides. They may be biosynthetically derived from one or several biosynthetic pathways [102].

Among stilbenes, *trans*-resveratrol (**1**) and piceatannol (**5**) are well-known phytoalexins or phytoalexin precursors that occur widely in plants; both are photosynthesis inhibitors [20,103].

The growth inhibitory and allelopathic activity of stilbenes **1**, **5**, **31** and **32** isolated from *S. maritimus* were investigated in different test systems (inhibition of 3PS leukaemia in mice, inhibition of potato crown gall tumors on discs of potato tubers, brine shrimp toxicity, fall army worm antifeedant activity, and growth inhibition of duckweed). The activities detected in these tests contribute to the ability of *Scirpus* species to survive and often dominate in various wetland plant communities [15].

Carexanes I (**27**) and K (**23**) significantly stimulated the root growth of *Dactylis hispanica*, *Petrorhagia velutina*, and *Phleum subulatum* at the highest (10^−4^ M) concentration, while pallidol (**40**) and distachyasin (**24**) were the most toxic on *P. subulatum*. All tested compounds (**13**, **16**–**27**, and **40**) inhibited or slightly stimulated the seedling growth with the only exception of *P. velutina* that was stimulated over 50% by the seco-carexane 25 [29]. Carexanes (**16**, **18**–**21**, **23**, **25**–**27**) were also tested for their phytotoxicity on the seeds of *Lactuca sativa*. The metabolites induced a weak decrease of germination (20%) of test organism. Furthermore, the compounds showed a stimulating effect on seedling growth. This effect was more evident on shoot elongation. Compound **18** stimulated shoot elongation at a lower concentration, while **21** increased shoot elongation in all tested concentrations [43].

#### 6.2.2. Pest Control by Acting on the Regulation of Insect Growth

Ecdysteroids are essential for insects in their physiological processes (e.g., in moulting). Phytoecdysteroids have the potential to disrupt physiological processes of susceptible insect species. Susceptible insect species would absorb such phytoecdysteroids into the hemolymph in an unregulated manner where these would bind to existing receptors and act to produce abnormal physiological situations during insect growth and development [104]. Based on these properties, phytoecdysteroids can be used for pest control.

*cis*-Miyabenol C (**43**), kobophenol B (**63**), and *cis*-miyabenol A (**64**), isolated from *C. pendula*, were found to antagonize the action of 20-hydroxyecdysone in *Drosophila melanogaster* with EC_50_ values 19, 37, and 31 µM, respectively, using a microplate-based B_II_ cell bioassay [30].

## 7. Side Effects of Stilbenes Occurring in Cyperaceae Species

Similarly to the pharmacological activities of stilbenes, the most information about their adverse effects is available in the case of resveratrol (1). Although many studies have indicated that 1 is a well-tolerated and safe compound in humans [105], some have reported on its toxic effects in vitro and in vivo [106,107,108]. These controversial effects of resveratrol (1) are due to its biphasic dose-dependent effects, which means that at low doses it has a stimulating effect associated usually with the beneficial (among others the antioxidant) effects, while at higher doses it possesses inhibitory properties resulting in the toxic effects (e.g., pro-oxidant feature) of this compound. In some cases, both effects can be advantageous, e.g., low concentrations can be useful in the prevention of cancer formation (chemopreventive) while higher doses can be used in the treatment of cancer (cytotoxic) [63,109]. All the toxic side effects (e.g., ulcerogenicity, renal toxicity, and detrimental cardiovascular effects) of **1** are mentioned to be related to its high-dosage-associated hormetic effects in vitro and in vivo [104,105,106,110,111,112,113].

In addition, it was shown that **1** interacts with several drugs. These interactions are harmful since, in most cases, they could attenuate the activities of these drugs [114]. It was reported that resveratrol (1) alters or inhibits the enzyme CYP3A4 [115] leading to possible alteration of the metabolism of a high percentage of marketed drugs. Furthermore, resveratrol was proven to inhibit the function and expression of drug transporters [like P-glycoprotein, multidrug resistance-associated protein 2 (MRP2), or organic anion transporters (OAT1/OAT3)], thus enhancing the bioavailability of certain drugs, e.g., nicardipine [116], methotrexate [117] and fexofenadine [118]. Other type of drug interactions were also detected [63], resveratrol (1) attenuating the effect of human immunodeficiency virus (HIV) protease inhibitors [119] and potentiating the effects of 3-hydroxy-3-methylglutaryl coenzyme A reductase (HMG-CoA reductase) inhibitors [120] and calcium channel agonists [121].

Additionally, long-term intake of resveratrol (**1**) can act as a thyroid disruptor and a goitrogen [122]. In case of other stilbenes, only limited data were published in the literature about their adverse effects.

## 8. Conclusions

According to the literature data, plants belonging to the family Cyperaceae are rich sources of stilbenoids. The presence of stilbenes in sedges is not restricted to any organ, but they can be found in underground parts and seeds in a higher ratio. Similarly to other stilbene-containing plant families, resveratrol (**1**) is the most common monomer in Cyperaceae species. Oligostilbenoids are composed of building block compounds of resveratrol (**1**) and piceatannol (**5**), which produces the fused skeletons of heterocyclic (forming a tetrahydrofurane ring) and bicyclic systems. Some *Carex* species accumulate a large amount (>1000 ppm) of oligostilbenoids, especially tetramers. It is interesting that up to now, tetrastilbenes were isolated only from *Carex* and *Kobresia* species. Prenylsubstituted stilbenes were isolated from sedges only in monomeric forms. Carexanes from *C. distachya* are unique tetracyclic stilbenoids originating from prenylated stilbenes by cyclization. Interestingly, besides prenyl-substituted stilbenes, prenyl substituted flavonoids were also detected in *S. nigricans*, proving that stilbenes and flavonoids arose in the same biosynthetic pathway. Cyperaceae species accumulate monomeric, di-, tri- and tetrameric stilbenes; among them monomers are substituted with hydroxy, methyl, methoxy, glucose, prenyl and carboxyl moieties, while in cases of di-, tri- and tetramers only hydroxy-substitution occurs.

As a result of the ethylene bridge, stilbenes can occur as *cis*- and *trans*-isomers, of which the *trans*-isomer (*E*) is the most common as it is more stable than the *cis* one. Different pathways lead to *cis*-*trans* isomerization, e.g., direct photoisomerization under solar or UV irradiation, or thermal isomerization. The same observation could be detected in case of Cyperaceae stilbenes; only two compounds were published with *cis* configuration [*cis*-miyabenol A (**64**) and *cis*-miyabenol C (**43**)]. In these cases, it was observed that if they were exposed to light, *trans*-isomerization was performed. Probably, this is the reason that much less *cis*-isomers are isolated.

In case of Cyperaceae stilbenoids, valuable biological activities were detected in pharmacological studies. The most promising ones are the antitumor [α-viniferin (**42**), nepalensinols A‒F (**45**–**47**, **57**–**59**)], antioxidant (**31**–**38**), antimicrobial [α-viniferin (**42**) and ε-viniferin (**30**)] and anthelmintic [*trans*-resveratrol (**1**) and α-viniferin (**42**)] effects. Monomer stilbenes have been studied much more intensely then oligomers. This is probably related to their higher abundance in nature and simple structure enabling their easier identification and further structural modification towards novel derivatives. Moreover, as stilbenoids are present in plants in mixtures with other polyphenols, their possible synergistic effects may be expected.

Some pharmacological results confirm the traditional uses of plants (e.g., use of *C. baccans* as an anthelmintic), but almost all data are derived from in vitro investigations, therefore, to certify the effectiveness of these plants in human therapy further investigations, especially in vivo and human studies are needed. Moreover, because of the controversial effects of resveratrol (**1**), the molecular mechanism of actions of bioactive stilbenes, isolated from sedges, need to be identified.

In conclusion, Cyperaceae species are promising sources of biologically active stilbenes, and hopefully even more research groups will deal with the phytochemistry, pharmacology, bioavailability and potential utilization of sedges.

## Figures and Tables

**Table 2:** Pharmacological activities of Cyperaceae extracts and isolated stilbenes.

Species	Extract/Compound	Method	Effect	Ref.
*Antiproliferative activity*				
*C. gynandra*, *C. folliculata*	**1**, **40**, **42**, **44**, **62**, **63**	Cell lines: HT-29, HCT-116 and Caco-2 (human colon tumor cell lines) and CCD-18Co (normal colon cell line) Positive control: resveratrol (**1**)	antiproliferative, α-viniferin (IC_50_ 6.6 μM on HCT-116 cells)	[31]
*C. rotundus*	racemates of **48**+**49**, **50**, **55**, **56**	human T-cell leukemia Jurkat cells	antiproliferative, IC_50_ values 27.4 µM (**48** + **49**), 40.5 µM (**50**), 26.4 µM (**55**) and 26.3 µM (**56**)	[9]
*K. nepalensis*	**45**–**47**, **57**–**60**	human DNA topoisomerase II Positive controls: etoposide, daunorubicin	topoisomerase II inhibitory activity IC_50_ values 0.30 µM (**45**), 0.02 µM (**57**), 7.0 µM (**46**), 14.8 µM (**47**), 11.7 µM (**58**), 5.5 µM (**59**), respectively. Etoposide (IC_50_ = 70 µM), daunorubicin (IC_50_ = 9.1 µM)	[36,51]
*Antioxidant activity*				
*C. longus*	methanol extract	DPPH	scavenging activity, SC_50_ = 22 µg/mL	[26]
	**1**, **5**, **31**–**38**, **40**	DPPH Positive control: ascorbic acid	SC_50_ = 2.8 µM (**32**), 8.2 µM (**31**), 2.8 µM (**32**) 4.6 µM (**33**), 9.3 µM (**34**), 4.3 µM (**35**), 5.0 µM (**36**), 3.2 µM (**37**), and 4.5 µM (**38**), 24 µM (**1**), 11 µM (**5**), 29 µM (**40**).	[26]
	**31**, **48**+**49**, **50**, **55**, **56**	DPPH Positive control: ascorbic acid	free radical scavenging activity, 65% at 5 µg/mL (**55**) 58% (**50**), 49% (**48**+**49**), 47% (**56**) and 37% (**31**), ascorbic acid (46%).	[9]
*C. dystachya*	methanol extract (root)	DPPH Positive controls: of ascorbic acid, α-tocopherol and butylated hydroxytoluene (BHT)	IC_50_ value 4.2 µg/mL (extract), 4.3 µg/mL (ascorbic acid), 5.1 µg/mL (α-tocopherol), 3.9 µg/mL BHT.	[54]
	**3**, **4**, **24**, **41**	superoxide radical, H_2_O_2_, NO, TBARS Positive control: ascorbic acid	NO radical scavenging activity: >80% for **3** and **4**, 58.5% for ascorbic acid and α-tocopherol, 62.2% for BHT. Radical scavenging activity of **24** against superoxide radical 60% at 0.5 mg/mL, 32% and 39% at 0.1 and 0.2 mg/mL (hydrogen peroxide), 10.7% at 0.5 mg/mL (NO radical), >59.0% at 0.5 mg/mL TBARS	[43,54]
*S. californicus*	**5**, **31**, **32**	xanthine oxidase	IC_50_ values 3.9 µM (**5**), 3.6 µM (**31**) and 6.0 µM (**32**)	[45]
*Anti-inflammatory activity*				
*C. humilis*	**42**	COX inhibition of PGH_2_ synthase Positive controls: indomethacin, resveratrol (**1**)	IC_50_ ~7 µM (**42**), ~5 µM (indomethacin), 25 µM (**1**)	[46]
*C. dystachya*	**16**, **22**, **27**	HspB-transfected human gastric epithelial (AGS) cells	Enhancement of the antioxidant response of AGS cells. Reduction of Keap-1 gene expression, and induction of NQO1 gene expression in AGS cells. Decrease of COX-2 gene expression in HspB-transfected AGS cells.	[42]
*Antiallergic activity*				
*C. longus*	methanol extract **1**, **5**, **36**–**38**	in vivo; ear passive cutaneous anaphylaxis (PCA) rat basophilic leukaemia (RBL-2H3) cells; *β*-hexosaminidase release inhibition Positive controls: tranilast, ketotifen fumarate	75% inhibition of PCA reactions at 500 mg/kg per os Inhibition of the release of β-hexosaminidase (IC_50_ values 17 µM (**1**), 24 µM (**5**), 96 µM (**36**), 84 µM (**37**), and 84 µM (**38**) in rat basophilic leukaemia (RBL-2H3) cells. IC_50_ values were 112 µM for tranilast, and 176 µM for ketotifen fumarate.	[26]
*Antibacterial activity*				
*C. fedia* var. *miyabei*	**30**, **44**, **64**, **65**	disc diffusion: 10, 50, 250 μg/disc (diameter 8 mm) Test bacteria: *Staphylococcus aureus*, *Bacillus subtilis*, *Escherichia coli*, *Cladosporium herbarum*, *Mucor mucedo*, *Aspergillus niger*, *Fusarium solani*, *Saccharomyces cerevisiae*	**64** showed antimicrobial activities against *S. aureus* and *B. subtilis* at a level of less than 10 µg/8 mm diameter paper disc.	[28]
*C. humilis*	**42**	drug-susceptible and -resistant strains of *Mycobacterium tuberculosis* (H37Rv), methicillin-susceptible *Staphylococcus aureus* (MSSA), methicillin-resistant *S. aureus* (MRSA) and methicillin-resistant *S. epidermidis* (MRSE) Combination: streptomycin, ethambutol	MIC values 4.6 µM for both strains in culture broth medium and 2.3‒4.6 µM inside macrophages and pneumocytes. An additive effect and partial synergy against *M. tuberculosis*, applying in combination with streptomycin and ethambutol. **42** did not show cytotoxicity in any of the cell lines tested up to a concentration of 147 µM (selectivity index >32). Inhibition of the proliferation of MSSA, MRSA and MRSE (MIC values 9.2–18.4 µM).	[56]
*C. dimorpholepis*	methanol extract **1**	enterohemorrhagic *Escherichia coli* (EHEC)	Inhibition of biofilm formation of *Escherichia coli* O157:H7 0.1 mg/mL >90% (extract) after 24 and 48 h The extract decreased the adhesion of EHEC cells to human colonic epithelial (HT-29) cells without affecting the viability of these cells Resveratrol (1) possessed anti-biofilm activity against EHEC at 10 µg/mL.	[53]
*C. pumila*	methanol extract **1**, **30**	*P. aeruginosa* PA14 and enterohemorrhagic *Escherichia coli* O157:H7 (EHEC)	Inhibition of biofilm formation of *Pseudomonas aeruginosa* PA14 and *Escherichia coli* O157:H7 at 0.1 mg/mL by 89% (extract) and at 10 µg/mL by 98% (**30**) without affecting planktonic growth.	[50]
*Anthelminthic activity*				
*Carex baccans*	root tuber extract **1**	in vivo; rats *Hymenolepis diminuta* Positive control: praziquantel *C. baccans* extract: 10, 25 and 50 mg/kg b.w. Resveratrol: 1141; 2282 és 4564 mg/kg b.w. 5 mg/ttkg prazikvantel EPG (egg per gram) value before and 1 week after treatment, and after 39 days	The root tuber extract at 50 mg/kg b.w., and its stilbene constituent **1**, at 4.56 mg/kg b.w. reduced EPG count (56.0% and 46.1%) of *Hymenolepis diminuta*, and decreased worm burden by 44.3% and 31.0%.	[13]
	**1**, **42**	*Raillietina echinobothrida* Positive control: praziquantel AChE, NO synthase Positive controls: Nω-nitro-l-arginine, pyridostigmine	Parasites ceased movement at 9.4, 11.4, and 0.2 h, followed by death at 23.7, 34.2, and 1.9 h, respectively. Significant decrease in the activity of acetylcholinesterase (46.1 and 65.9%) and nitric oxide synthase (61.2% and 55.0%) were detected in comparison with the controls Nω-nitro-l-arginine (29.6%) and pyridostigmine (63.6%). The anthelmintic effect of these compounds is mediated through inhibition of two vital enzymes.	[47]
*Antidiabetic activity*				
*C. baccans*	Ethyl acetate extract **42**	α-glucosidase, α-amylase	The ethyl acetate extract of *C. baccans* and compound **42** inhibited α-glucosidase [IC_50_ values 31.8 µg/mL (extract), and 6.8 µg/mL (**42**)] and α-amylase [IC_50_ values 421.1 µg/mL (extract), and 282.9 µg/mL (**42**)] enzymes.	[52]
*Vasorelaxant activity*				
*C. appressa* var. *virgata* *C. cuprina* *C. eragrostis*	**5**, **30**, **32**, **55**, **61**	in vivo; arginase enzyme ex vivo; rat aorta ring	Compounds **5**, **30**, **32**, **55**, and **61** inhibited significantly arginase enzyme with IC_50_ values 12.6, 27.8, 22.6, 12.2 and 25.3 µM, respectively. Increased NO bioavailability vasorelaxant effect	[25]
*Allelopathic activity*				
*C. dystachya*	**13**, **16**–**27**, **40**	*Phleum subulatum*, *Dactylis hispanica* and *Petrorhagia velutina* seeds	Compounds **23** and **27** significantly stimulated the root growth of *D. hispanica*, *P. velutina* and *P. subulatum* at a concentration 10^−4^ M. **24** and **40** were the most toxic on *P. subulatum*. All compounds inhibited or slightly stimulated the seedling growth with exception of *P. velutina* that was stimulated over 50% by **25**.	[29]
*C. dystachya*	**16**, **18**–**21**, **23**, **25**–**27**	phytotoxicity *Lactuca sativa* seeds	All compounds induced a weak decrease of germination (20%) of *Lactuca sativa* seeds. All compounds showed a stimulating effect on seedling growth, especially on shoot elongation. **18** stimulated shoot elongation at a lower concentration. **21** increased shoot elongation in all tested concentrations.	[43]
*Ecdysteroid antagonistic activity*				
*C. pendula*	**43**, **63**, **64**	in vitro; *Drosophila melanogaster* BII cell bioassay	Compounds **43**, **63**, and **64** antagonized the action of 20-hydroxy-ecdysone in *Drosophila melanogaster* with EC_50_ values 19, 37, and 31 µM, respectively.	[30]
